# Describing the perceptions of student nurses regarding barriers and benefits of a peer-mentorship programme in a clinical setting in KwaZulu-Natal

**DOI:** 10.4102/hsag.v24i0.1118

**Published:** 2019-10-08

**Authors:** Zanele P. Mlaba, Waheedha Emmamally

**Affiliations:** 1 School of Nursing and Public Health, University of KwaZulu-Natal, Durban, South Africa

**Keywords:** student mentorship, barriers, benefits, clinical settings, peer mentorship, peer mentor, peer mentee

## Abstract

**Background:**

Novice student nurses encounter challenges when making the transition to clinical learning because of the complex and unpredictable nature of clinical settings. A selected campus initiated a peer-mentoring programme based on research findings, which revealed that student nurses were inadequately mentored in clinical settings because of mentors experiencing work overload, time and resource constraints, staff shortage and patient-care demands.

**Aim:**

The aim of this study was to describe the perceptions of student nurses regarding barriers and benefits of a peer-mentorship programme in a clinical setting.

**Setting:**

The study was conducted in a clinical setting in KwaZulu-Natal.

**Methods:**

A quantitative, descriptive design was used, whereby data were collected through questionnaires. A total of 56 mentors (third- and fourth-year nursing diploma student nurses) and 94 mentees (first- and second-year nursing diploma students) participated in the study.

**Results:**

The mentors highlighted insufficient practice opportunities because of the short duration of the placement, time and resource constraints, and the simultaneous mentoring of too many students as barriers, while the mentees highlighted the reluctance of mentors to fulfil their roles and lack of dedication and unfriendliness as barriers to effective mentorship. The benefits of being a mentor were self-achievement, enhancement of skills and acquiring of positive work ethics, while the major mentee benefits included experiencing less anxiety, adapting to the clinical environment easily and being less intimidated in the clinical setting.

**Conclusions:**

This study revealed that both mentors and mentees perceived peer-mentoring programmes as important to their clinical growth. However, these programmes require formative evaluations to address negative perceptions of student mentees and mentors and to identify challenges faced by them.

## Introduction

Clinical practice plays a pivotal role in the personal, professional and clinical development of student nurses. The Minimum Requirements Guide Concerning the Teaching of Students in the Programme Leading to Registration as a Nurse (General, Psychiatry and Community) and Midwifery of 1992, as amended in 1994, stipulated that students should be exposed to 400 h of clinical practice (South African Nursing Council 1992). According to the curriculum of the KwaZulu-Natal College of Nursing (KZNCN), first-year student nurses spend a minimum of 100 h in clinical placement. The purpose of the clinical practice is to provide student nurses with meaningful clinical learning opportunities relevant to their level of training.

Literature indicates that the initial clinical experience can be stressful and intimidating, and may cause significant fear, anxiety, uncertainty and a feeling of abandonment for the novice student nurse (Moscaritolo 2009; Payton et al. 2013; Purfeerst 2011). These experiences are attributed to the unpredictable and complex nature of the clinical environment and are exacerbated in student nurses who have no prior clinical experience. High levels of anxiety can affect students’ clinical performance and may pose a threat to executing their tasks adequately because of poor coping skills (Li et al. 2010; Moscaritolo 2009; Purfeerst 2011).

A study by Mhlaba (2011) revealed that students perceived professional nurses’ mentorship in the clinical setting to be inadequate. Reasons cited included the heavy workloads of professional nurses, time and resource constraints, staff shortages and high patient-care demands. The KZNCN in response to the conclusions of Mhlaba (2011) recommended a structured support programme that would use innovative interventions to address student’s clinical challenges. Peer mentoring was identified by KZNCN as one of the strategies that would facilitate professional growth and development of student nurses in clinical practice. It was believed that a student peer-mentoring programme, which addressed the academic, psychosocial and clinical support of novice student nurses, could play a vital role in retaining students while meeting learning outcomes successfully. This resulted in the implementation of a peer-mentoring system in KZNCN to provide students with experiential learning under the mentorship of peers.

Peer mentoring has been defined as an interactive process that enables nursing skill acquisition in a safe and non-threatening environment that emphasises practice and feedback (Sibiya, Ngxongo & Beepat 2018). According to Stone, Cooper and Cant (2013), peer mentoring is a student-centred learning approach that stimulates active student participation, where student nurses take responsibility for their own learning. It fosters critical thinking, psychomotor and clinical skills, cognitive development and academic gains, all of which are essential characteristics for meeting present and future health challenges. Dennison (2010) asserted that peer mentorship encompasses supportive relationships formed between equals and helps to prevent problems in busy, stressful practice settings. In support of this, Jokalainen et al. (2011) stated that adequate student support and positive clinical experiences might increase student enthusiasm and retention. Peer mentoring is viewed as an effective method of promoting positive interaction between learners of different cultural backgrounds, and improving academic and clinical achievement, interpersonal relationships and attitudes towards the clinical environment (Botma, Hurter & Kotze 2013). Robinson and Neimer (2010) added that peer mentoring enhances a range of skills, including teamwork, collaboration, reflection and communication skills, which are important requirements in nursing.

According to Mhlaba (2011), unlike other countries, such as the United Kingdom, the United States and Australia, the regulating body (SANC) in South Africa does not have formal mentoring programmes for preparing professional nurses to mentor student nurses. Professional nurses, as part of their supervisory and teaching functions, historically mentor student nurses in the clinical setting. However, because of the national shortage of professional nurses, the task of student nurse supervision has been assigned to graduates and inexperienced nurses (Tshabalala 2011). In addition, nursing education is confronted by many challenges, including increasing class sizes, rising student numbers, rising competency requirements, decreasing numbers of lecturers and limited clinical placement areas (Dennison 2010; Kaphagawani & Useh 2013; Mhlaba 2011). These challenges compromise clinical supervision and teaching of student nurses, with peer mentoring therefore providing additional support to students in clinical settings. Stone et al. (2013) proposed that senior student nurses could effectively teach and supervise the junior staff, thus decreasing the demands on lecturers and professional nurses.

Joubert and De Villiers (2015) highlighted that the success and effectiveness of support programmes of student nurses in clinical settings must be determined through feedback from mentors and mentees. Robinson and Neimer (2010) asserted that despite research indicating notable advantages of peer-mentorship programmes in the clinical area, the lack of programme evaluation and mentor and mentee feedback impacts the success of these programmes. Notably, the effectiveness of the peer-mentoring programme implemented by KZNCN has not been evaluated since its inception in 2012. Furthermore, no study has described the student nurses’ perceptions regarding the barriers to and benefits of the peer-mentorship programme.

The aim of this study was therefore to describe the perceptions of student nurses regarding barriers to and benefits of a peer-mentorship programme in a clinical setting in KwaZulu-Natal.

## Research method and design

### Research approach

A non-experimental descriptive study design was adopted whereby data were collected by means of questionnaires.

### Research population and sample

The study population included 210 student nurses registered for the 4-year comprehensive diploma in general nursing who had participated in the peer-mentoring programme. Students were purposively selected based on their exposure to the peer-mentoring programme. Inclusion criteria for the mentee population included nursing students who were older than 18 years and who were either first- or second-year students with exposure to the mentorship programme. The inclusion criteria for the mentor population were third- and fourth-year student nurses who were ‘mentors’, as a requirement for the Ethos and Professional Practice module in their third year of training. The mentor sample consisted of 80 respondents and the mentee sample comprised 110 respondents. In consultation with a statistician, the study included all willing respondents to cater for attrition.

## Setting

The study was conducted on the KZNCN campus, which is affiliated to a district hospital where students are placed for their clinical practice.

### Reliability and validity measures

Two questionnaires (one for the mentee cohort and one for the mentor cohort) were developed by the researcher and an expert in nursing education to address the aim of the study. Questions that measure perception focus on how an individual’s perceptions may affect an individual’s behaviour or opinions (Lavrakas 2008). In this study, the perceptions of the mentee and mentor cohorts regarding the benefits of and barriers to the mentorship programme were measured by eliciting their responses to close-ended statements.

Section A of the questionnaires focussed on demographic constructs of the mentor and mentee, including ethnicity, gender, age and involvement in peer mentoring. Section B focussed on constructs related to the barriers and benefits of the peer-mentorship programme.

Both the questionnaires were subjected to the scrutiny of experts in nursing education and research for content validity. Cronbach’s alpha showed that both questionnaires reached the acceptable reliability of 0.721 and 0.880, respectively. Construct validity was determined by conducting a pilot study on a sample of 20 students (10 mentors and 10 mentees) representing 10% of the study sample to ensure readability of the questionnaires.

### Data collection and analysis

The questionnaires administered by the researcher took approximately 30 min to be completed. The questionnaires required respondents to either agree or disagree with the statements addressing the barriers to and benefits of peer mentoring.

Descriptive statistics were used to describe and summarise the collected data and measures, with frequency and percentage distributions presented in tables and graphs. The latest version of computerised statistical software package (SPSS 22) was used to organise and analyse the data.

### Ethical consideration

Ethical clearance was obtained from the Department of Health (reference number: 296/15 KZ_2015RP16_333) and the Humanities and Social Sciences Ethics Committee (reference number: HSS 1348/015M).

## Results

A total of 150 questionnaires were completed. Fifty-six of the 80 (70%) mentors and 94 (85%) of the 110 mentees completed the questionnaires, providing a response rate of 79%.

### Demographic data of respondents

The socio-demographics of the 56 mentors and 94 mentees combined were as follows: 61% black people, 1.5% white people, 31.5% Indians and 6% mixed race. The gender distribution of mentors was as follows: 28.6% males and 71.4% females, while the mentees’ cohort had 21.3% males and 78.7% females. Concerning age distribution, the mentor cohort was in the 20–24 years age range, while the mentees were in the age range of 18–19 years (7.4%; *n* = 7). The youngest respondent was 18 years old. The mentor cohort had a lower percentage of respondents who were in the 20–24 years age range compared to the mentee cohort (51.8%; *n* = 29 mentors; 68.1%; *n* = 64 mentees). The mentors had 19.2% (*n* = 11) and the mentee cohort had 9.6% (*n* = 9) of respondents who were in the 30–35 years age range and older. Approximately, 46.6% (*n* = 26) of mentors had been involved in the mentoring programme as both mentors and mentee.

### Barriers to peer mentoring in the clinical setting

The statements regarding barriers to peer mentoring in the clinical setting as perceived by the mentor and mentee cohorts are arranged in descending order of agreement.

#### Mentor barriers

Majority of mentor respondents perceived that a busy clinical setting (92.9%), lack of clinical staff support (91.1%) and insufficient practice opportunities (91.1%) were the major barriers to peer mentoring in the clinical setting. Fewer mentor respondents (58.9%) perceived the age gap between mentors and mentees to be a barrier to peer mentoring (Table 1).

**TABLE 1:** Mentors’ perceptions of barriers to peer mentoring (*n* = 56).

Barriers	Agree		Disagree	
	*N*	%	*N*	%
1. Performing mentoring duties in a very busy clinical setting with very sick patients	52	92.9	4	7.1
2. Lack of support from clinical staff members	51	91.1	5	8.9
3. Insufficient practice opportunities for the students because of the short duration of the placement	51	91.1	5	8.9
4. Working with limited equipment and other resources	51	91.0	5	9.0
5. Conflict of interest because of the demands of the nursing programme and from the peer mentors as well	50	89.3	6	10.7
6. Lack of understanding of the programme requirements	45	80.3	11	19.7
7. Inadequate time available to attend to both the mentees and patients	45	80.3	11	19.6
8. Poor preparation to carry out the role of peer mentor	43	76.8	13	23.2
9. Lack of recognition of the demand of the role of peer mentoring by nurse educators	42	75.0	14	25.0
10. Mentoring too many students at the same time	36	64.3	20	35.7
11. Assisting a learner whose skills levels are below the expected standard	34	60.7	22	39.3
12. Cross-cultural and cross-gender mentoring create discomfort	34	60.7	22	39.3
13. Too wide an age gap between peer mentor and peer mentee	33	58.9	23	41.1

#### Mentee barriers

Many mentee respondents (89.4%) perceived inadequate resources and discrepancy between what was taught in class and what was implemented in the clinical settings (84%) as the main barriers to peer mentorship. Only 57.4% of the mentee respondents perceived demographic differences to be a barrier to peer mentorship in the clinical setting (Table 2).

**TABLE 2:** Mentees’ perceptions of barriers to peer mentoring (*n* = 94).

Barriers	Agree		Disagree	
	*N*	%	*N*	%
1. Working with limited equipment and other resources	84	89.4	10	10.6
2. Discrepancy between what is taught in class or in simulation and what is actually implemented in the clinical setting causes anxiety and confusion	79	84.0	15	16.0
3. Mentors who seem to be uncertain about their knowledge and actions make students nervous and anxious	73	77.7	21	22.3
4. Reluctance of mentors to fulfil their roles; mentors who are not dedicated and unfriendly	73	77.7	21	22.3
5. Lack of support from clinical staff members	65	69.1	29	30.9
6. Too wide an age gap between peer mentor and peer mentee	56	59.6	38	40.4
7. Poor preparation to carry out the role of peer mentoring	54	57.4	40	42.6
8. Cross-cultural and cross-gender mentoring create discomfort	54	57.4	40	42.6

## Benefits of peer mentoring in the clinical settings

The statements regarding benefits of peer mentoring in the clinical settings as perceived by the mentor and mentee cohorts are arranged in descending order of agreement.

### Mentor benefits

All mentors perceived that their confidence increased during mentoring and that nurses have a professional responsibility to teach students and peers (Table 3). Around 98.2% of mentor respondents perceived benefits of enhancement of personal gratification, early exposure to being a role model, and promotion of personal and professional development. Fewer mentor respondents (89.2%) perceived feeling important, respected and valued as the benefits of peer mentoring.

**TABLE 3:** Mentors’ perception of benefits of peer mentoring (*N* = 56).

Benefits	Agree		Disagree	
	*N*	%	*N*	%
1. Acting the role of a peer mentor increased my confidence	56	100.0	-	-
2. Nurses have a professional responsibility to teach students and peers	56	100.0	-	-
3. Facilitating and aiding learning and development of a less experienced nurse enhances personal gratification	55	98.2	1	1.8
4. Mentoring provided an early exposure to being a role model	55	98.2	1	1.8
5. Providing support and encouragement to a junior nurse promoted my personal and professional development	55	98.2	1	1.8
6. Engaging in peer mentoring helps to gain an opportunity to review knowledge and stay current with skills	54	96.4	2	3.6
7. Peer mentoring enabled the application of principles of teaching and learning	53	94.6	3	5.4
8. Acting the role of peer mentor enabled the development of teaching and leadership skills	53	94.6	3	5.4
9. Peer-mentoring experience prepared me for my professional nurse’s role	53	94.6	3	5.4
10. Peer-mentoring experience was time and effort well spent	52	92.9	4	7.1
11. Providing psychosocial support to mentees makes the mentors feel important, respected and valued	50	89.2	6	10.7

### Mentee benefits

All mentee respondents (100%) agreed that the role of a peer mentor increased their confidence, self-esteem and autonomy. Mentee respondents also perceived benefits of mentors giving helpful feedback (98.9%), which in turn enabled easy adaptation to the clinical environment (96.8%). Fewer respondents (92.6%) perceived that enhancement of communication skills was a benefit of per mentorship (Table 4).

**TABLE 4:** Mentees’ perceived benefits of peer mentoring (*N* = 94).

Benefits	Agree		Disagree	
	*N*	%	*N*	%
1. Self-confidence, independence and ability to perform clinical skills are increased	94	100.0	-	-
2. The feedback received from my peer mentor is from a student’s viewpoint, and therefore more honest, reliable and helpful than from the instructor	93	98.9	1	1.1
3. Makes adapting to the clinical environment easy	91	96.8	3	3.2
4. Less anxiety is experienced when performing nursing skills in the presence of peers	90	95.7	4	4.3
5. Peer mentoring helped in integration of theory and practice	90	95.7	4	4.3
6. Makes one to be less intimidated and more comfortable	89	94.7	5	5.3
7. Teaching is an important role of nurses	89	94.7	5	5.3
8. Approaching a peer mentor for assistance is easier than approaching the instructor	88	93.6	6	6.4
9. Communication with my peer mentor is freer than with the instructor	87	92.6	7	7.5
10. The peer mentor was supportive when I was performing a nursing skill	86	91.5	8	8.5
11. When a peer mentor teaches a clinical skill, interaction and collaboration with other students increases more than when my instructor teaches it	85	90.4	9	9.6

## Discussion

Barriers that challenged mentor availability included increased clinical workload, insufficient time available to attend to both the mentees and patients, mentoring too many students at the same time and performing mentoring duties in a very busy clinical setting with very sick patients. In support of these findings, student nurses in Ravanipour, Bahreini and Ravanipour (2015) mentioned that smaller workloads would enable them as mentors to teach each other more accurately and effectively. The current study revealed that mentors indicated being overwhelmed by the mentoring responsibilities, as they did not have protected time away from clinical duties to fulfil their mentoring role effectively. Some studies (Mhlaba 2011; Tshabalala 2011; Van Graan et al. 2016) found that as a result of staff shortage, students were being treated as workforce preventing them from being exposed to a wider range of experiences necessary to meet their learning outcomes. Respondents also indicated a lack of understanding of programme requirements and inadequate preparation to carry out the mentoring role. This is in line with studies (Dennison 2010; Joubert & De Villiers 2015; Stone et al. 2013) asserting that the deliberate and extensive preparation and orientation to become mentors contributed to the uniqueness of the programme. Likewise, Al-Hamdan et al. (2014) argued that if the nursing school and the health care organisation do not recognise the mentors’ needs, it makes them feel unrewarded and ill prepared. Shortage of material resources, such as equipment and supplies, to execute nursing care was identified by 91% of the respondents. Mogale (2011) concurs that lack of equipment prevents students from undertaking nursing procedures, thus missing out on teaching and learning opportunities. The availability of all necessary equipment in the clinical setting serves a dual function of promoting quality nursing care and facilitating effective demonstration of practical skills (Mogale 2011). Mentors indicated being challenged by having to assist learners whose skill levels were below the expected standard and who also displayed a lack of initiative and motivation. Similar findings are noted in Joubert and De Villiers (2015) where peer mentors felt that there were instances where mentees did not meet the mentors’ expectations of enthusiasm for learning.

It was evident that the mentees perceived that there existed a discrepancy between what was taught in class and what was actually implemented in the clinical setting. This precipitated anxiety and confusion compounded by mentors who seemed to be uncertain about their knowledge and actions. The reluctance of mentors to fulfil their roles, lack of dedication and unfriendliness challenged the effectiveness of mentoring (Kaphagawani & Useh 2013). According to Ferguson (2010), peer mentors must demonstrate confidence in word and deed to foster mentee trust. They must keep abreast of new knowledge and evidence-based practice so that they are able to supervise juniors with authority of knowledge. A few students in this study indicated that the support and involvement of unit managers in peer mentoring was lacking. Researchers (Hodgson & Scanlan 2013; Joubert & De Villiers 2015) stress that the nursing profession needs to commit to and embrace the concept of mentoring to provide novice student nurses with supportive learning environments, in which they can grow and flourish. The authors added that it remains the responsibility of the peer mentoring coordinator from the college to inform the unit managers about the peer-mentorship programme so that the mentors and mentees are supported and clinical learning opportunities are made available.

Mentees in the study perceived that they experienced less anxiety, adapted to the clinical environment easily and were less intimidated in the clinical settings. The majority indicated that it was easier to approach a peer mentor for assistance than the instructor. Several studies (Dennison 2010; McKenna & French 2011; Teatheredge 2010) acknowledge that the constant support that the mentees get from their mentors enables them to practise nursing skills because they presume that the clinical area is a safe environment in which they are able to learn through experience. Stone et al. (2013) also support that mentoring provides a more relaxed, less intimidating, learning experience than sessions conducted by professional nurses. El-Sayed, Metwally and Abdeen (2013) noted contradictory findings where students who were taught by peers felt less motivated to practise a skill and interact with peers. The research findings indicated that peer mentoring facilitated the mentees’ ability to relate to and apply theory.

This study found that the mentor respondents indicated enhanced sense of achievement and confidence in their knowledge and skill level; reflection on their own learning and on their own practice; gaining an opportunity to review knowledge and update current skills as major benefits. These findings validate a previous study by Denison (2010). Peer mentors acquire skills and gain intrinsic rewards by contributing to the mentees’ education and helping them to succeed in such a challenging profession (Gisi 2011; Joubert & De Villiers 2015; Kurtz, Lemley & Alverson 2010). Peer mentors also highlighted that the programme enabled the application of principles of teaching and learning. Rosenau et al. (2015) pointed out that as a result of engaging in peer mentoring, students discovered that they have a potential for teaching and that they want to teach in the future. This study also revealed that enacting the role of a peer mentor enabled the development of teaching and leadership skills. This is argued by Henning, Weidner and Marty (2008), who indicated that nursing students involved in peer teaching and learning improve their psychomotor skills and their overall clinical knowledge. In support of this, Dennison (2010) and Gisi (2011) stated that by participating in a mentoring programme and offering skills and support, the mentor gains experience as a leader, as a teacher and is able to enhance his or her communication skills.

## Limitations of the study and recommendations

The study findings are limited to a population sample of one nursing campus of the KZNCN; therefore, the perceptions of student nurses on the barriers and benefits of peer-mentorship programmes cannot be generalised to other campuses or universities, both nationally and internationally.

Recommendations of the study include the following:

- Appointment of a programme and liaison coordinator, responsible for the training of mentors and mentees for their respective roles.
- Designing a pre-orientation package, which includes information on the mentorship programme and mentor profiles.
- Summative and continuous formal evaluations of the programme should be conducted to ensure effectiveness and sustainability.
- Further research on this topic is recommended whereby there should be a control group to verify if peer mentoring has positive effects and identify barriers for mentors and mentees.

## Conclusion

While some students highlighted negative perceptions, which impeded the effectiveness of a peer-mentoring programme, there is evidence which confirms that peer mentorship in contemporary nursing practice remains integral to students’ clinical experiences. It is expected that the recommendations made will mitigate and correct the negative perceptions. The study findings revealed that peer mentoring enhances adaptation and facilitates the transition of first-year student nurses to clinical learning. Nursing is a caring profession and peer mentors by virtue of being nurses should be compassionate to new student nurses to help them in their journey of transitioning from a novice to an competent clinician. Further research is needed to evaluate the effectiveness of the changes that will be implemented to reduce negative perceptions.
